# Npn-1 Primes Limbs for Motion

**DOI:** 10.1371/journal.pbio.1001023

**Published:** 2011-02-22

**Authors:** Rachel Jones

**Affiliations:** Freelance Science Writer, Welwyn, Hertfordshire, United Kingdom

During neural development, the axons of sensory and motor neurons must extend over long distances—meters in some animals—to reach the most distant parts of the limbs. The axons of these neurons adhere tightly together to form spinal nerves that project over these distances to their peripheral targets. This process, called fasciculation, controls axon outgrowth and guidance. However, the underlying signaling pathways that mediate this process are not well understood.

Two molecules, semaphorin 3A (Sema3A) and neuropilin-1 (Npn-1), have been implicated in axon–axon interactions that mediate the segregation of axons in the olfactory system (a process that is similar to fasciculation of axons that innervate the limbs), and it has also been suggested that they are involved in the fasciculation of motor axons before they grow into the forelimb. Npn-1 is expressed in both spinal motor neurons and sensory neurons of the dorsal root ganglia, making it a potential mediator of interactions between the axons of these neurons.

In this issue of *PLoS Biology*, Huber and colleagues have used conditional mouse genetics to investigate whether Npn-1 plays a role in the segregation and fasciculation of sensory and motor axons before they innervate peripheral targets. First, they generated mice in which Npn-1 expression was abolished in most motor neurons but not in sensory neurons. Lack of Npn-1 in motor neurons led to extreme defasciculation of motor axons and to dorsal–ventral guidance errors in the axon trajectories in the limb. Sensory axons, by contrast, were unaffected.

As the next logical step, they then generated mice in which only sensory neurons of the dorsal root ganglia lacked Npn-1 expression. In these mice, both sensory and motor axons projecting to the limbs were strongly defasciculated and showed aberrant trajectories. Closer examination of the spinal nerves showed that defasciculated motor axons always followed sensory axons in these mice.

After exiting the spinal cord and dorsal root ganglia, motor and sensory axons converge in a region called the plexus and are sorted into bundles according to their targets. In mice that lacked Npn-1 in motor neurons, defasciculation occurred only after motor axons had passed through the plexus. By contrast, in mice that lacked Npn-1 in peripheral sensory neurons, both motor and sensory axons were defasciculated before they reached the plexus as well as within and beyond it, with sensory fibers leading motor axons. These results support a role for Npn-1 in mediating the mutual dependence of sensory and motor axons during proper circuit wiring.

In another experiment, the authors used genetic techniques to partially eliminate motor neurons. Mice with few motor neurons had reduced and abnormal sensory projections, although sensory axons projected normally towards the plexus region. On the other hand, elimination of sensory neurons caused defasciculation of motor axons as well as the remaining sensory axons before they reached the plexus.

The results of these experiments support a model in which motor axons are required for sensory neurons to route through the plexus region, with sensory axons subsequently able to project into the limb on their own. Motor projections also rely on sensory axons for correct fasciculation and guidance. The role of Npn-1 and the importance of axon–axon interactions seem to vary depending on the location, offering important insight into the mechanisms of peripheral limb innervation.

**Huettl R-E, Soellner H, Bianchi E, Novitch BG, Huber AB (2011) Npn-1 Contributes to Axon-Axon Interactions That Differentially Control Sensory and Motor Innervation of the Limb. doi:10.1371/journal.pbio.1001020** [Fig pbio-1001023-g001]

**Figure pbio-1001023-g001:**
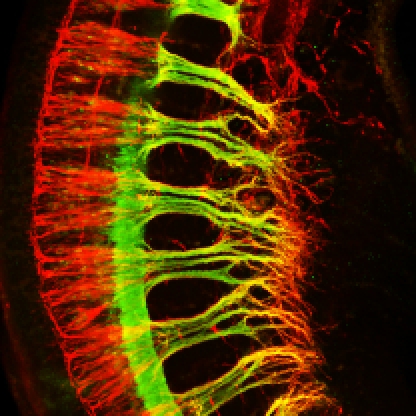
**Growing sensory (red, neurofilament staining) and motor axons (green, Hb9::eGFP) in the brachial plexus of the forelimb of a mouse E10.5 embryo. Removal of the axon guidance receptor neuropilin-1 from sensory neurons results in breaking of the tight couping between sensory and, motor axons and defasciculation of sensory and, surorisingly, also motor trajectories.** Image: Rosa-Eva Huettl, Helmholtz Zentrum München – German Research Center for Environmental Health, Germany.

